# Neuropsychological and biopsychosocial evolution, therapeutic adherence and unmet care needs during paediatric transplantation: study protocol of a mixed-methods design (observational cohort study and focus groups) – the TransplantKIDS mental health project

**DOI:** 10.3389/fpsyg.2024.1308418

**Published:** 2024-02-21

**Authors:** Jessica Garrido-Bolton, Margarita Alcamí-Pertejo, Rocío de la Vega, Francisco Hernández-Oliveros, Antonio Pérez-Martínez, María Fe Bravo-Ortiz, Eduardo Fernández-Jiménez

**Affiliations:** ^1^Department of Psychiatry, Clinical Psychology and Mental Health, La Paz University Hospital, Madrid, Spain; ^2^Department of Personality, Evaluation and Psychological Treatment, University of Málaga, Málaga, Spain; ^3^Instituto Biomédico de Málaga – IBIMA Plataforma Bionand, Málaga, Spain; ^4^Department of Pediatric Surgery, La Paz University Hospital, Madrid, Spain; ^5^Department of Pediatric Hemato-Oncology, La Paz University Hospital, Madrid, Spain; ^6^Hospital La Paz Institute for Health Research (IdiPAZ), Madrid, Spain; ^7^Department of Pediatrics, Universidad Autónoma de Madrid (UAM), Madrid, Spain; ^8^Department of Psychiatry, Universidad Autónoma de Madrid (UAM), Madrid, Spain; ^9^Centro de Investigación Biomédica en Red de Salud Mental (CIBERSAM), Instituto de Salud Carlos III, Madrid, Spain; ^10^Faculty of Social Sciences and Communication, Universidad Europea de Madrid, Madrid, Spain

**Keywords:** neuropsychological outcomes, biopsychosocial model, organ transplantation, allogeneic hematopoietic transplantation, children/adolescents, observational cohort design, focus groups

## Abstract

The present article describes the protocol of a mixed-methods study (an observational cohort design and focus groups), aimed to examine neuropsychological functioning and other biopsychosocial outcomes, therapeutic adherence and unmet care needs in paediatric population undergoing solid organ or allogeneic hematopoietic transplant during the pre- and post-transplant phases. Following a multi-method/multi-source approach, neuropsychological domains will be comprehensively measured with objective tests (SDMT, K-CPT 2/CPT 3, TAVECI/TAVEC, WISC-V/WAIS-IV Vocabulary and Digit Span subtests, Verbal Fluency tests, Stroop, ROCF, and TONI-4); ecological executive functioning, affective and behavioral domains, pain intensity/interference, sleep quality and therapeutic adherence will be assessed through questionnaires (parent/legal guardians-reported: BRIEF-2 and BASC-3; and self-reported: BASC-3, BPI, PROMIS, AIQ and SMAQ); and blood levels of prescribed drugs will be taken from each patient’s medical history. These outcomes will be measured at pre-transplant and at 4-weeks and 6-months post-transplant phases. The estimated sample size was 60 patients (any type of transplant, solid organ, or hematopoietic) from La Paz University Hospital (Madrid, Spain). Finally, three focus group sessions will be organized with patients, parents/guardians, and transplant clinicians (*n* = 15, with 5 participants per group), in order to qualitatively identify unmet care needs during the pre-, and post-transplant stages of the process. The study protocol was registered at ClinicalTrials.gov (NCT05441436).

## 1 Introduction

Healthcare professionals and researchers focus on optimizing clinical and patient-reported transplant outcomes in children and adolescents. In doing so, studies must elucidate when the introduction of diagnostic tests and interventions, frequently very time-consuming, is more cost-effective, whether in the pre-, peri-, and/or post-transplant phases (Annema et al., 2023).

The intervention of Mental Health Liaison teams in this clinical population has been shown to be fundamental in the evaluation of the suitability of living donor candidates, assessing the recipient’s understanding of the transplant, treating psychopathological comorbid conditions, and in the intervention with the patients’ relatives (Amatya et al., 2021). Moreover, psychosocial predictors of medical regimen adherence must be monitored by Mental Health professionals throughout the whole process, both in the pre- and post-transplantation phases, to prevent negative medical outcomes (e.g., increased allograft rejection, loss of the organ, retransplantation, and death) (Killian et al., 2018). However, although these lines of intervention have been, to some extent, covered in some children hospitals, the scientific evidence accumulated to date identifies other areas of action that every reference hospital for paediatric transplantation should protocolize. In particular, these include standardized assessment of patients throughout the entire process (pre-, post-transplant and follow-up phases) on biopsychosocial (quality of life, emotional and behavioral problems) and neuropsychological domains (Jesse and Haver, 2022), which will be the main objective of this project.

In particular, the promotion and evaluation of the mental health of patients (Di Giuseppe et al., 2020; Ünay et al., 2020; Proli et al., 2021), as well as of their relatives, must be an essential axis of action in the clinical care of the paediatric population undergoing transplantation, which has become especially evident during the recent health emergency situation resulting from the COVID-19 pandemic (Zhao et al., 2020). Importantly, patients’ mental health problems (such as, anxiety, depression, distress, behavioral problems) and family conflicts are modifiable psycho-social predictors of non-adherence to medications (Killian et al., 2018) and, particularly relevant, suffering depression increases risk of mortality among patients after organ transplant (Dew et al., 2015). Moreover, family conflicts have been associated with worse evolution of the mental health of children and adolescents after transplantation (Sinatora et al., 2020). Therefore, it is essential to identify dysfunctions at the family and other interpersonal level in this population (Chardon et al., 2020).

Moreover, as mentioned previously, other functional and clinical indicators of the paediatric population in the process of transplantation or in those already transplanted should also be systematically incorporated into hospitals’ protocols for these patients, such as neuropsychological evaluations. In this regard, scientific literature confirms that this clinical population presents outstanding needs both at the level of global intellectual functioning and specific neurocognitive domains, both in transplant recipients of different solid organs (Alonso and Sorensen, 2009; Urschel et al., 2018; Gold et al., 2020; Karakizlis et al., 2022; Østensen et al., 2022) and of hematopoietic progenitors (Fahnehjelm et al., 2018; Wu et al., 2022).

Specifically, paediatric solid organ transplant recipients have demonstrated overall worse performance in attentional control, working memory, processing speed and executive functioning in general (Cushman et al., 2020). Moreover, verbal comprehension and perceptual reasoning are also found to be significantly below those of healthy children. Some studies indicate that when compared to the reference group, twice as many children in the transplant group obtain total IQ scores of less than 70 (Fredericks et al., 2014). Common risk factors for these neurocognitive difficulties include malnutrition, infections, long hospital admissions, and altered environmental interactions that reduce age-normative stimulation. Moreover, each patient subgroup (according to organ transplant type) had specific reasons for cognitive risks during the transplantation process. For example, among patients with renal disease, the accumulation of waste products from impaired renal function can affect cognitive outcomes. Similarly, liver patients could suffer cognitive consequences through the accumulation of ammonia as well as chronic malnutrition. For patients undergoing heart transplantation, there is a cognitive risk associated with cardiopulmonary bypass and circulatory arrest if they have undergone surgical repair of cardiac abnormalities, as well as an increased risk of ischemic hypoxia throughout the disease course (Reed-Knight et al., 2015).

Likewise, cognitive impairment is an established sequela of allogeneic hematopoietic transplantation. Amongst others, it has been noted that these patients have significant deficits in learning, processing speed, verbal fluency, memory, and executive functioning. There are multiple potential contributors to cognitive impairment in this clinical population, including chemotherapy cytotoxic effects, increased incidence of autoimmune phenomena, and a chronic proinflammatory state affecting brain health, even in the absence of frank Graft-Versus-Host-Disease (GVHD). This population is also more vulnerable to post-transplant infections, which can potentially affect cognitive performance (Buchbinder et al., 2018).

Although past research has demonstrated neurocognitive difficulties in paediatric patients’ post-transplantation phase, there are limited data examining patients’ cognitive functioning prior to transplantation (Reed-Knight et al., 2015). Therefore, this study proposes a comprehensive neuropsychological protocol to be implemented before and after transplantation to measure changes over time. This assessment, together with the evaluation of other biopsychosocial domains, will allow for the early detection of high-risk clinical conditions at the mental health level as well as the identification of peri-surgical neurocognitive disorders (Kaustov et al., 2021). Similarly, early identification of these deficits is essential to be able to activate potential educational adaptations or cognitive rehabilitative interventions, which will be optimally effective if implemented in the critical periods of child neurodevelopment (Karakizlis et al., 2022).

In addition, it is now strongly recommended that the planning of psychological interventions should follow a co-design approach with those involved in the care process (Palermo et al., 2021; Annema et al., 2023). In this sense, throughout this project, several focus groups will be carried out with patients and their families, as well as with their medical/surgical professionals of reference, to identify the unmet care needs throughout the pre-, peri-, and postoperative phases. These findings will allow for the design of future psychological interventions for surgical prehabilitation (i.e., to prepare the patient to be in the best possible state before surgery).

Finally, the most recent scientific evidence focuses on identifying which factors mediate the relationship between several patient-reported outcomes (e.g., pain, anxiety, depression and sleep disorders) and their adherence to prescribed treatments in the paediatric population, identifying executive functioning as a variable of special interest (Caes et al., 2021).

Despite the evidence cited above and to the best of our knowledge, no study has been conducted in Spanish paediatric populations with the aim of identifying potential neurocognitive and other biopsychosocial concerns, as well as unmet care needs throughout the entire transplantation process.

## 2 Objectives

The objectives of the present study were as follows:

− Examine neuropsychological and other biopsychosocial outcomes in paediatric population during the pre- and post-transplant phases at 4 weeks and 6 months.
− Identify unmet care needs of patients and their parents/guardians in the pre- and post-transplant phases.
− Compare neuropsychological and other biopsychosocial outcomes by age groups and type of transplant.
− Compare pre- and post-transplant therapeutic adherence by age groups and type of transplant.
− Analyze the variables that mediate the relationship between several patient-reported outcomes and treatment adherence.
− Identify possible peri-surgical neurocognitive disorders.

### 2.1 Hypotheses

The hypotheses of the present study are listed below and are based on clinical experience as well as on the available scientific literature (Caes et al., 2021):

*H1:* Improvements in some neuropsychological domains (e.g., processing speed and executive functioning) will be observed between the pre- and post-transplant phases.

*H2:* Improvement in some parent- (or guardians-) and patient-reported outcomes (e.g., emotional-behavioral problems and personal adjustment) will be observed between the pre- and post-transplant phases.

*H3:* Patients under cardiac and lung transplantation will suffer poorer neuropsychological outcomes and more emotional-behavioral problems, both in the pre- and post-transplantation phase, than patients under kidney transplantation.

*H4:* Patients under hematopoietic transplantation will suffer poorer neuropsychological and more emotional-behavioral problems, both in the pre- and post-transplantation phase, than patients under solid organ transplantation.

*H5:* Older patients will show poorer therapeutic adherence levels than younger patients.

*H6:* Executive functioning will mediate the relationship between several patient-reported outcomes (e.g., pain, anxiety, depression and sleep disorders) and therapeutic adherence.

## 3 Methods and analysis

### 3.1 Study design

This project follows a mixed-methods approach, hence the present study protocol will follow the SPIRIT 2013 guidelines (Chan et al., 2013) adapted to the STROBE statement (von Elm et al., 2007), for the observational study; and to the COREQ statement (Tong et al., 2007), for the qualitative study (focus groups).

### 3.2 Selection of participants

The potential participants will be all those children and adolescents already included (or in the imminent phase of being included) in the waiting list for a transplant of any solid organ (single or combined), or allogeneic hematopoietic from La Paz University Hospital (Madrid, Spain). The estimated sample size was 60 patients (for any type of transplant, organ, or hematopoietic). Recruitment will last from November 1, 2022, until June 2024.

For this purpose, a non-probabilistic technique of incidental sampling (or purposive sampling technique) will be used in this study according to the following inclusion and exclusion criteria listed below, based on other related studies:

#### 3.2.1 Inclusion criteria

- Age between 6 and 18 years.
- Females and males.
- Spanish as mother tongue or a very high level of Spanish to understand their participation in the study as well as to be able to complete the measurement instruments.

#### 3.2.2 Exclusion criteria

- Diagnosis of an autism spectrum disorder, severe sensory/motor limitations, or acquired brain damage.
- Presence of a severe uncontrolled comorbid disease, independent of the disease motivating the transplant.

### 3.3 Ethical considerations

Firstly, this study will be carried out in accordance with the principles established in the Helsinki declaration, strictly respecting confidentiality and the requirements of Spanish (14/2007, of July 3, 2007, on Biomedical Research; and Organic Law 3/2018, of December 5, 2018) and European data protection regulations.

Secondly, all those legally responsible for the patients will receive an information sheet about the study, will be able to resolve their questions about the study and will freely give their informed consent so that minor patients can participate in the present study. It will be emphasized at all times that participation in this research is completely voluntary and that refusal to participate in the study will not have any unfavorable repercussions for the patient in their care process, neither will abandoning the study at any given phase. In those cases in which the parents of the patient are divorced and there is no way of obtaining consent from both of them, the participation of the minor in this research will be rejected.

Clinical data will be kept separate from identification data, and the databases will be encrypted and stored on computers used exclusively for this project. All records will be maintained in compliance with the precepts established in current legislation on the protection of personal data (Organic Law 3/2018, of December 5, 2018). The persons responsible for processing the research data will only do so in accordance with the purpose stated on the informed consent form. Once the purpose of this study is accomplished, all personal data will be destroyed, as will any documentary support containing the personal data incorporated into the study.

This study was approved by the Research Ethics Committee of La Paz University Hospital (Madrid, Spain).

### 3.4 Variables and instruments

The variables measured in this project, the instruments used and the informants who will complete each measure are detailed below:

- Socio-demographic (sex, age, patient’s level of studies, parents’ level of studies) and clinical variables (current pharmacological treatment—active substance and dose). These data will be collected with an initial survey created *ad hoc* for the present study.
- Processing speed. The Symbol Digit Modalities Test (SDMT) (Smith, 2002) will be administered to patients.
- Visual sustained attention/vigilance. The Kiddie Continuous Performance Test, second edition (K-CPT 2) (Conners, 2006) will be administered to children aged 4 to 7 years, and the Continuous Performance Test, third edition (CPT 3) (Conners, 2014) to children aged 8 and above. This test computes several scores, as follows: Detectability, Omissions, Commissions, Perseverations, Hit Reaction Time (HRT), HRT Standard Deviation, Variability, HRT Block Change and HRT Inter-Stimulus Interval Change.
- Immediate and delayed verbal memory. A Spanish test based on the California Verbal Learning Test will be administered to patients. Specifically, the Spain-Complutense Verbal Learning Test for Children (TAVECI) (Benedet et al., 2017) will be administered to children aged 3 to 16 years; and the version for older patients (TAVEC) (Benedet and Alejandre, 2014) will be applied to adolescents aged 16 to 18 years. This test allows calculating a wide range of scores regarding the verbal memory performance, and the following scores will be specifically used in this study: Immediate Recall, Short Delay Free Recall, Short Delay Cued Recall, Long Delay Free Recall, Long Delay Cued Recall, Long Delay Recognition, Semantic clustering strategies, Serial clustering strategies, False positives, and Discriminability.
- Semantic memory. The Vocabulary subtest of the Wechsler Intelligence Scale for Children, fifth edition (WISC-V) (Wechsler et al., 2015) will be administered to patients up to the age of 16.11 years, and the adult version, fourth edition (WAIS-IV) (Wechsler, 2012), will be applied to patients up to 18 years.
- Verbal working memory and cognitive flexibility. The Digit Span subtest of the WISC-V (Wechsler et al., 2015) or the WAIS-IV version (Wechsler, 2012) will be administered to patients. Three neurocognitive domain-specific scores will be considered from the three differentiated parts of the task (Forward, Backward, and Sequencing subscales), as well as the global score (Digit Span) as a measure of cognitive flexibility (Wechsler et al., 2015).
- Verbal fluency. The Verbal Fluency Test (TFV) (Portellano Pérez and Martínez Arias, 2020) will be administered to patients. This test calculates the following eight scores: Phonological fluency, Semantic fluency, Excluded-letter fluency, Total Fluency Index, Errors in phonological fluency, Errors in semantic fluency, Errors in excluded-letter fluency and Total errors.
- Ability to inhibit cognitive interference. The Stroop Colour Word Test (SCWT) (Golden, 2020) will be administered to patients. Four scores can be computed from this test, such as: the Word reading (W), the Colour naming (C), the Colour-word (CW) and Resistance to Interference.
- Visuo-constructive organizational ability and immediate and delayed visual memory. The Rey-Osterrieth Complex Figure (ROCF) test (Rey, 2003) will be applied to patients. This test generates four scores: a copy score (accuracy in terms of visual–spatial constructional ability), the time required to copy the figure, and two delayed-recall scores (at 3 and 30 min).
- Non-verbal Intelligence. The Test of Non-verbal Intelligence, fourth edition (TONI-4) (Brown et al., 2019), using both parallel forms (A and B), will be administered to patients.
- Ecological Executive Functioning. The Behavior Rating Inventory of Executive Function, second edition (BRIEF-2) (Maldonado-Belmonte et al., 2017), in its parent-reported version, will be completed by the patient’s parents (or legal guardians).
- Emotional and behavioral problems. The Behavioral Assessment System for Children and Adolescents, third edition (BASC-3) (Reynolds and Kamphaus, 2020) will be administered, both the parent-reported version, by the patient’s parents (or legal guardians), and the self-reported version, by the patient. This questionnaire covers the personal (e.g., adaptive skills, resiliency, externalizing, and internalizing problems) and interpersonal/social domains (e.g., school problems, relationships with parents, interpersonal relations) of each patient.
- Pain intensity and interference. The Brief Pain Inventory (BPI) (Badia et al., 2003) will be used as a self-reported measure by patients. This questionnaire includes questions (from 0 to 10 referring to the last seven days) covering two dimensions: pain intensity (worst and mildest pain in the week, average pain, and current pain), and pain interference (general activity, mood, walking, school, sleep, as well as enjoyment of life).
- Sleep problems. The PROMIS Pediatric Sleep Disturbances Short Form (Forrest et al., 2018) will be used as a self-reported questionnaire by patients. It is a 4-item questionnaire that assesses problems of sleep onset and maintenance in the last 7 days.
- Insomnia. The Adolescent Insomnia Questionnaire (AIQ) (Bromberg et al., 2020) will be used as a self-reported measure by patients. It is a 13-item questionnaire that covers three domains: Sleep Dissatisfaction and Impairments, Sleep Onset, and Sleep Maintenance.
- Adherence to treatment. The Simplified Medication Adherence Questionnaire (SMAQ) (Ortega Suárez et al., 2011) will be used to evaluate therapeutic adherence as a self-reported measure. Also, blood levels of the prescribed drugs (mainly immunosuppressants) will be used to objectively evaluate therapeutic adherence, which will be extracted from the clinical history of each patient.

Table 1 summarizes the measurement instruments used, the domains covered by each instrument, as well as the informants who will complete each measure.

**Table 1:** Variables and instruments of the project: dimensions measured, test and informants.

Dimensions measured	Test	Ages	Informant
-Socio-demographic data. -Medication: active ingredient and dose.	Survey created *ad hoc* for this project.	6–18 years old	Parent/guardian
**Neuropsychological domains**			
Processing speed	*-Symbol Digit Modalities Test* (SDMT) (Smith, 2002).	6–85 years old	Patient
Visual sustained attention/vigilance	*-Kiddie Continuous Performance Test, 2nd edition* (K-CPT 2) (Conners, 2006). *-The Continuous Performance Test, 3rd edition* (CPT 3) (Conners, 2014).	4–7 years old 8–83 years old	Patient
Immediate and delayed verbal memory	*-España-Complutense Verbal Learning Test for Children* (TAVECI) (Benedet et al., 2017). *-España-Complutense Verbal Learning Test* (TAVEC) (Benedet and Alejandre, 2014).	3–16 years old 16 – adults	Patient
Semantic memory	*-Vocabulary of the Wechsler Intelligence Scale for Children-V* (WISC-V) (Wechsler et al., 2015). *-Vocabulary of the Wechsler Intelligence Scale for Adults-IV* (WAIS-IV) (Wechsler, 2012).	6–16:11 years old 16–90 years old	Patient
Verbal working memory and cognitive flexibility	*-Digit Span of the Wechsler Intelligence Scale for Children-V* (WISC-V) (Wechsler et al., 2015). *-Digit Span of the Wechsler Intelligence Scale for Adults-IV* (WAIS-IV) (Wechsler, 2012).	6–16:11 years old 16–90 years old	Patient
Verbal fluency	*-Verbal Fluency Test* (TFV) (Portellano Pérez and Martínez Arias, 2020).	6 years old – adults	Patient
Ability to inhibit cognitive interference	*-Stroop Colour Word Test* (SCWT) (Golden, 2020).	6–85 years old	Patient
Visuo-constructive organization/planning and immediate and delayed visual recall	*-Rey-Osterrieth Complex Figure* (ROCF) (Rey, 2003).	6–80 years old	Patient
Non-verbal abstract reasoning	*-Test of Non-verbal Intelligence- 4* (TONI-4), parallel forms A and B (Brown et al., 2019).	6–79 years old	Patient
-Global executive function -Behavioral regulation -Emotional regulation -Cognitive regulation	*-Behavior Rating Inventory of Executive Function, 2nd edition* (BRIEF-2) (Maldonado-Belmonte et al., 2017).	5–18 years old	Parent/guardian
**Mental health, pain, sleep problems and therapeutic adherence**			
*Parent-reported version:* *-Adaptive skills*. *-Behavioral symptom index*. *-Externalizing problems*. *-Internalizing problems*. *Self-reported version:* *-Personal adjustment*. *-Inattention/Hyperactivity*. *-Internalization problems*. *-School problems*. *-Emotional symptoms index*.	*-Behavioral Assessment System for Children and Adolescents − 3* (BASC-3) (Reynolds and Kamphaus, 2020).	3–18:11 years old 8–18:11 years old	Parent/guardian Patient
-Pain intensity. -Pain interference.	*-Brief Pain Inventory* (BPI) (Badia et al., 2003).	6 years old – adults	Patient
Quality of sleep	-*PROMIS Pediatric Item Bank*. *Sleep Disturbance – Short Form 4a* (Forrest et al., 2018).	5–17 years old	Patient
-Dissatisfaction with sleep and deterioration. -Sleep onset. -Sleep maintenance.	-*Adolescent Insomnia Questionnaire* (AIQ) (Bromberg et al., 2020).	11–18 years old	Patient
-Taking prescribed medication. -Application of other prescribed guidelines. -Attendance at clinical consultations.	*Simplified Medication Adherence Questionnaire* (SMAQ) (Ortega Suárez et al., 2011).	6–18 years old	Patient
Adherence to prescribed treatments	Blood levels of prescribed drugs (mainly immunosuppressants), which will be taken from each patient’s medical history.	6–18 years old	Patient

### 3.5 Procedure

The fieldwork for this study will take place at La Paz University Hospital (Madrid, Spain) and it will be conducted by clinical psychologists trained in neuropsychological testing (JGB and EFJ).

First of all, patients and their parents (or legal guardians) will be briefly informed about this study by medical/surgical professionals and, if they agree, they will be referred to the Psychiatry, Clinical Psychology and Mental Health Unit at the Children hospital. This Mental Health team will inform the patients and/or their legal guardians, in a more exhaustive manner, about the objectives of the present study. An initial screening will be carried out to examine compliance with the inclusion/exclusion criteria and, if so, they will be given the information and informed consent forms. After authorization and consent to participate in this study, they will be sent electronic links to complete the battery of questionnaires, in which they will be identified with an anonymous code in order to preserve their identity under the strictest confidentiality; and, subsequently, the neuropsychological testing will begin. The neuropsychological assessment will be carried out in person and individually with each patient; while the digitalised assessment will require 4 types of links across the different platforms, from REDCap (Harris et al., 2009), Hogrefe TEA Ediciones and Pearson Clinical, for the self- and parent/legal guardians-reported questionnaires. Post-transplant evaluations will take place at 4 weeks and 6 months after transplantation. In these two post-transplant phases, the same type of tests and questionnaires described in the previous phase will also be administered, although with a shorter battery at 4 weeks. At each stage of the neuropsychological evaluation, tests will be administered during a single session. Sessions during the pre-transplant and the 6-months follow-up phases will have a duration of approximately one and a half to maximum 2 h (including a 10-min break). Testing at 4 weeks after transplantation will be of a shorter duration, around 45 min. After each assessment, the clinical psychologists will enter the data into the project database.

This study follows a mixed-methods approach: a quantitative methodology following an observational cohort design, based on standardized tests and questionnaires; as well as a qualitative methodology, based on focus groups.

On the one hand, the quantitative evaluations will take place in both pre- and post-transplantation phases. Specifically, the assessment in the pre-transplant phase will take place, when possible, within 0–6 months prior to the transplantation, as recommended by expert consensus (Kaustov et al., 2021). The post-transplantation phases will take place at 4 weeks and 6 months after the surgery.

Additionally, the patients’ parents (or legal guardians) will also have to authorize, in an informed manner, the participation of their children, as well as themselves in focus group sessions to qualitatively identify unmet care needs during the pre-, and post-transplant phases. Another focus group will also involve medical/surgical professionals of transplant recipients. There will be a total of 3 group sessions lasting up 90 min each (or until arriving at theoretical sufficiency), one session per participant type (patients, parents/guardians, and clinicians), 5 participants each group (*n* = 15 in total). All these focus groups will follow a biopsychosocial theoretical framework. Specifically, each focus group will begin indicating the group norms (confidentiality/privacy, not interrupting others’ turn to speak and not judging others’ comments). Afterwards, the session will continue with a first part of open questions about what aspects would have changed or improved in the health care, both for the patients and their parents, during the whole transplantation process. Subsequently, the second part of the focus group will pose specific questions about biomedical, psychological and social aspects that would have improved throughout the transplantation process and were not spontaneously addressed in the first part of each focus group. The sessions will be conducted by videocall, audio-recorded and automatically transcribed, respecting the confidentiality of the information at all times. The sessions will be facilitated by a member of the research team, different from the PI of this project (EFJ) to avoid biases (the PI knows most transplant clinicians, patients and their parents/legal guardians). The facilitator will be a psychologist, female, with Master’s Degree credentials. The automatically-generated transcriptions will be reviewed by the group facilitator present in the session in order to: (1) change the name of the participants by a code, and (2) correct any transcription mistakes. The recordings will be kept until it has been verified that the transcription process has been carried out correctly. Transcripts will not be returned to participants for comments. If needed, in anticipation that more relevant information may be collected, participants will be offered the possibility of individual in-depth interviews after the focus group. These interviews will be conducted under the same above-mentioned standards.

Finally, the results from this project will be disseminated and published in different scientific media and documents, both written (articles in specialized journals, posters at conferences) and oral (presentations at conferences).

The protocol of this study was registered at clinicaltrials.gov on July 1, 2022 (NCT05441436).

**Figure 1:**
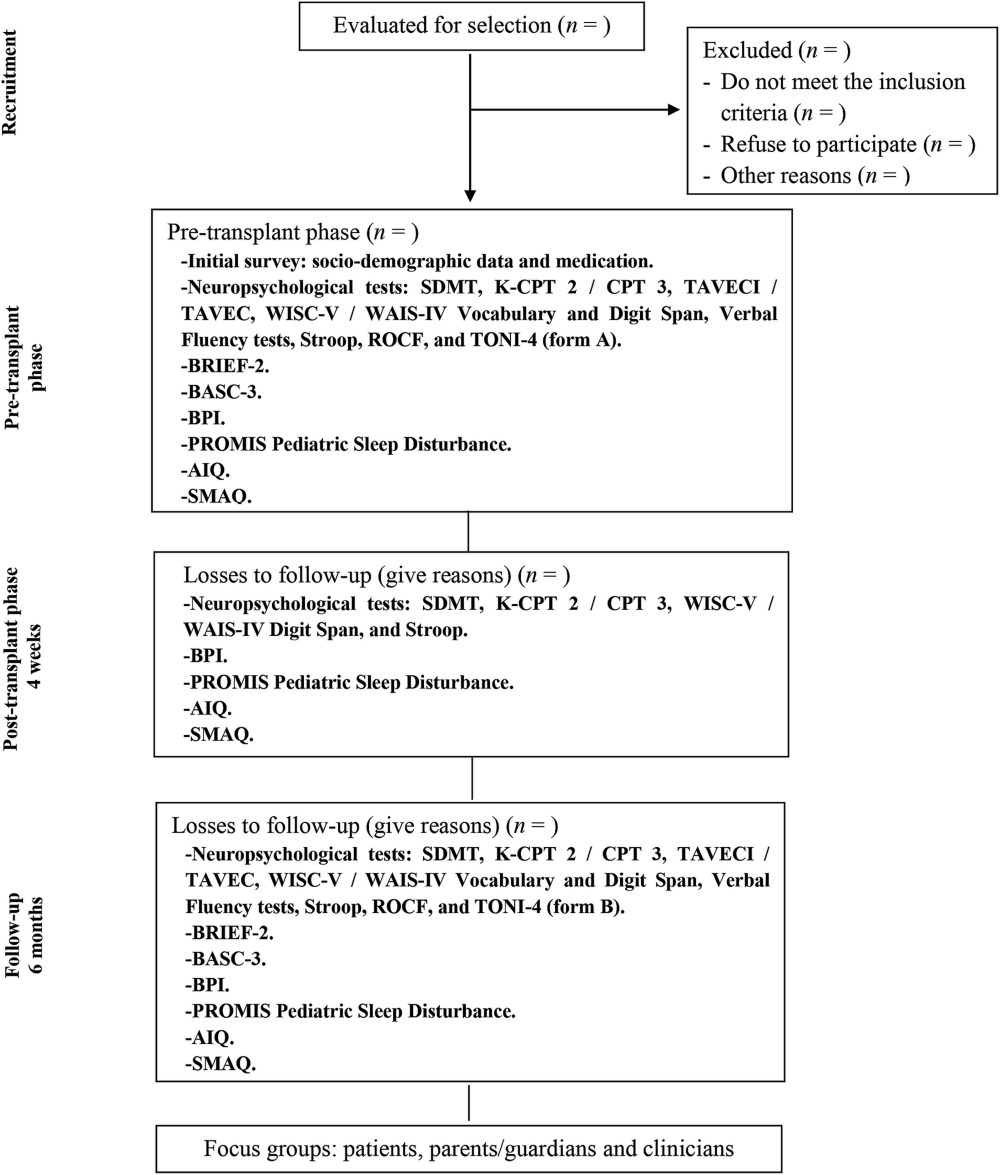
Flow diagram for study design. Study phases and outcome measures.

Figure 1 lists the phases of the study, as well as the variables and instruments in each phase.

### 3.6 Statistical analyses

The data will be processed primarily using the statistical software IBM SPSS Statistics 29, among others, and mainly the following analyses will be performed:

First, descriptive analyses will be carried out for all variables to identify possible errors or outliers in dataset. Then, zero order correlation analyses will be computed to determine the degree of association between variables; and partial correlation analyses will be conducted to examine its association excluding the effect of other confounding variables.

Additionally, as main statistical analysis, a mixed Analysis of Covariance will be performed to evaluate the main effects between groups (2 types of transplant: solid organ and haematopoietic) and within groups (time variable with 3 levels: pre-, post-transplantation at 4-weeks and 6-months follow-up) as well as potential interactive effects, excluding the effect of confounding variables. *P*-value adjustments for multiple comparison will be performed. In turn, other analytical strategies will be used to examine time series such as latent grow models. Finally, innovative statistical analyses based on multiple linear regression will be developed to examine the relative weight of the different predictor variables on the criterion variables, already published elsewhere (Fernández-Jiménez and Arnett, 2015).

All these statistical analyses will be complemented with the appropriate effect size indexes for each statistical test (e.g., *f*, omega-square and *R^2^* indexes) (Cohen, 1992). Additionally, Reliable Change Index (RCI) will be computed in order to determine the real change of each individual score between measurements over time (Kiselica et al., 2023).

Since the main statistical analysis in this study will be a mixed Analysis of Covariance (2 × 3), with a statistical power = 0.95 and a risk/significance level of alpha = 0.05, to detect at least a moderate effect size (*f* = 0.25) on the outcome variables included, a minimum sample size of 36 patients will be required according to GPower 3.1.9.4 software.

Regarding the qualitative study of this project (focus groups), thematic analysis techniques will be used, following the Clarke and Braun’s procedures (Clarke and Braun, 2021), in order to synthesize the themes (major and minor) related to the unmet care needs during the transplantation process. Themes will be derived from the data and not identified in advance. Two data coders will independently code the transcripts. Any disagreement will be resolved by consensus.

## 4 Discussion

This study aims to examine neuropsychological functioning and other biopsychosocial outcomes, therapeutic adherence and unmet care needs in paediatric population undergoing solid organ or allogeneic hematopoietic transplantations during the pre- and post-transplantation phases. The scientific literature encourages the incorporation of specific measures for mental health concerns as these can impact quality of life, morbidity and mortality in this population (Penner et al., 2022). Moreover, studies have confirmed that this clinical population presents outstanding needs related to impairment in specific neurocognitive domains. Therefore, given the wide range of neurocognitive deficits that have been identified in the paediatric population exposed both to solid organ transplantation and hematopoietic transplantation, this project has designed a comprehensive assessment protocol, covering the most common potential neuropsychological problems in this clinical population, such as intelligence, processing speed and attention, verbal and visual memory, and executive functioning measures (Reed-Knight et al., 2015; Buchbinder et al., 2018).

This project aims to fill the gap in the scientific literature regarding the comparison in patients’ neurocognitive performance and biopsychosocial domains before and after a transplantation process in order to assess change, prevent negative outcomes, and/or orientate adequate interventions.

Also, the results and conclusions derived from this project will be directly transferred to routine clinical practice and real-world patients. As mentioned above, the use of standardized measures (neuropsychological tests and biopsychosocial questionnaires) by the Mental Health team during the entire transplantation process will allow the early detection of potential neuro−/psychosocial problems, as well as the implementation or prescription of the adequate specific intervention (therapeutic or preventive) that may be required at an early stage (Karakizlis et al., 2022). For example, family disfunctions can be identified during any stage of the transplantation process so that an early intervention can be applied by Mental Health professionals (Cushman et al., 2019; Liu et al., 2020; Tadros et al., 2020). Consequently, this is expected to have a positive impact on the prognosis of the neurocognitive and mental health problems detected.

In short, the results of the pre- and post-transplant biopsychosocial and neuropsychological assessments will help to guide future clinical or educational interventions, if necessary, as well as to plan other activities to strengthen the patient’s resources, always with the aim of improving their personal, family, social and school adaptation. In the event that parents/legal guardians so request, clinical reports will be issued with recommendations that will help the patient’s educational centres to guide potential adaptations (curricular, assessment, or methodological) according to pedagogical criteria.

Among the methodological strengths of this study is the multi-method/multi-source approach, which includes not only self-/parent-reported questionnaires, but also objective neuropsychological tests and blood levels of prescribed drugs, thus avoiding common method bias (Podsakoff et al., 2012). These measures will constitute a multidomain set of outcomes that includes variables relevant to transplant recipients, their caregivers, and health professionals for decision making (Annema et al., 2023). However, our study presents several limitations. First, we will use a non-random sampling technique that increases the probability of some degree of collider bias and limits the generalizability of the findings. Second, because of the observational nature of the design, sound causal inferences cannot be drawn. Third, given the modest number of patients from the paediatric population undergoing a transplantation process, intergroup comparisons between types of solid organ transplants will be limited. Fourth, due to the clinical setting in which this project is implemented, other neurocognitive domains focused more specifically on school performance, such as reading or writing, will not be addressed. Last, attrition during post-transplantation or follow-up phases could bias the results because patients suffering more severe medical complications could be excluded from assessments, during the prefixed measurement times in this study.

Finally, the protocolisation of the assessment and monitoring of these clinical populations aims to create a working dynamic, of greater coordination between departments in our hospital, that will last beyond the years of this project, increasing the awareness among other clinicians about the potential neurocognitive and psychological needs of the patients and their relatives during the transplantation process.

## 5 Conclusion

A protocolised plan such as the one proposed in this project will allow the children hospital of La Paz University Hospital to improve the attention and care of all the participants in the transplant process: the patients, their families and the reference professionals.

Ultimately, this project aims to create a structured and permanent clinical care programme for children and adolescents undergoing transplantation, as other countries have been developing, given that the biopsychosocial repercussions suffered by these patients and their families continue for years after transplantation. Finally, the results will be disseminated in a clear and transparent manner, allowing for other healthcare centres to use them (Kikuchi and Kamibeppu, 2015; Bastos Tavares et al., 2022).

## Ethics statement

The standards of good clinical practice and the ethical principles for research involving human beings established in the Declaration of Helsinki and its subsequent revisions will be met at all times. This study was approved by the Research Ethics Committee of La Paz University Hospital (Madrid, Spain) (institutional code: PI-5223). Legal guardians of patients (or, when applicable, patients of legal age) will provide their written informed consent to participate in this study.

## Author contributions

JG-B: Conceptualization, Data curation, Investigation, Project administration, Resources, Supervision, Writing – original draft, Writing – review & editing. MA-P: Conceptualization, Methodology, Project administration, Supervision, Writing – original draft, Writing – review & editing. RV: Conceptualization, Methodology, Supervision, Writing – original draft, Writing – review & editing, Formal analysis, Funding acquisition, Validation. FH-O: Conceptualization, Methodology, Supervision, Writing – review & editing, Investigation. AP-M: Conceptualization, Formal analysis, Funding acquisition, Investigation, Methodology, Supervision, Writing – review & editing. MB-O: Conceptualization, Funding acquisition, Investigation, Project administration, Supervision, Writing – review & editing. EF-J: Conceptualization, Data curation, Formal analysis, Funding acquisition, Investigation, Methodology, Project administration, Resources, Supervision, Writing – original draft, Writing – review & editing, Validation.
